# Prosody-Based Sound-Emotion Associations in Poetry

**DOI:** 10.3389/fpsyg.2018.01284

**Published:** 2018-07-25

**Authors:** Maria Kraxenberger, Winfried Menninghaus, Anna Roth, Mathias Scharinger

**Affiliations:** ^1^Language and Literature, Max-Planck-Institut für empirische Ästhetik, Frankfurt, Germany; ^2^Department for Psycholinguistics and Didactics of German, Goethe-Universität Frankfurt am Main, Frankfurt, Germany; ^3^Phonetics Research Group, Department of German Linguistics, Center for Mind, Brain and Behavior, Philipps-Universität Marburg, Marburg, Germany

**Keywords:** emotional prosody, poetry, emotion perception, joy, sadness, articulation

## Abstract

Conveying emotions in spoken poetry may be based on a poem's semantic content and/or on emotional prosody, i.e., on acoustic features above single speech sounds. However, hypotheses of more direct sound–emotion relations in poetry, such as those based on the frequency of occurrence of certain phonemes, have not withstood empirical (re)testing. Therefore, we investigated sound–emotion associations based on prosodic features as a potential alternative route for the, at least partially, non-semantic expression and perception of emotions in poetry. We first conducted a pre-study designed to validate relevant parameters of joy- and sadness-supporting prosody in the recitation, i.e. acoustic production, of poetry. The parameters obtained thereof guided the experimental modification of recordings of German joyful and sad poems such that for each poem, three prosodic variants were constructed: one with a joy-supporting prosody, one with a sadness-supporting prosody, and a neutral variant. In the subsequent experiment, native German speakers and participants with no command of German rated the joyfulness and sadness of these three variants. This design allowed us to investigate the role of emotional prosody, operationalized in terms of sound-emotion parameters, both in combination with and dissociated from semantic access to the emotional content of the poems. The findings from our pre-study showed that the emotional content of poems (based on pre-classifications into joyful and sad) indeed predicted the prosodic features pitch and articulation rate. The subsequent perception experiment revealed that cues provided by joyful and sad prosody specifically affect non-German-speaking listeners' emotion ratings of the poems. Thus, the present investigation lends support to the hypothesis of prosody-based iconic relations between perceived emotion and sound qualia. At the same time, our findings also highlight that semantic access substantially decreases the role of cross-language sound–emotion associations and indicate that non-German-speaking participants may also use phonetic and prosodic cues other than the ones that were targeted and manipulated here.

## Introduction

Poetry is widely regarded as a genre in which semantic and formal components of language are interrelated in a particularly meaningful way (Jakobson, 1960; Menninghaus et al., 2017). According to literary theory, this results in a pronounced “palpableness” of form (Jakobson, 1960) and, most notably, of the sound patterns in poetry (Shklovsky, 1919/2012; Jakobson and Waugh, 1979/2002). Theoretical reflections as well as empirical studies have repeatedly highlighted the relation between the sound of a poem and the perception of emotion by its readers (e.g., Valéry, 1958; Fónagy, 1961; Jakobson and Waugh, 1979/2002; Tsur, 1992; Whissell, 2002, 2011; Schrott and Jacobs, 2011; Aryani et al., 2016).

Several studies have advanced the idea of a relation between the frequency of occurrence of segmental units, i.e., of certain phonemes or phoneme classes, and emotion perception in poetry. For instance, one study reported that nasals are more frequent in Old Egyptian lamentations and ballads written by the German poet J. W. von Goethe, whereas plosives are more frequent in Old Egyptian and Goethean hymns (Albers, 2008). Similarly, another study reported that native speakers rated German, Chinese, Russian, and Ukrainian poems with a relatively high frequency of nasals as sad, and poems with a high frequency of plosives as happy/joyful (Auracher et al., 2010). A more recent study (Kraxenberger and Menninghaus, 2016a), however, could not replicate Auracher et al's (2010) findings and consequently questions a consistent frequency effect. Thus, it seems that the frequently assumed nexus between sound and emotion perception in poetry (e.g., Valéry, 1958; Fónagy, 1961; Tsur, 1992; Whissell, 2002, 2011) might not consistently be driven by the frequency of occurrence of certain phoneme classes.

We therefore tested the hypothesis that acoustic, suprasegmental features of emotional prosody might rather be a possible reason of this nexus. Furthermore, we compared poems that have previously been classified as either joyful or sad (Kraxenberger and Menninghaus, 2016b). This allowed us to examine both bottom-up information from emotional prosody and top-down information from semantic content. Based on previous research, we exclusively focused on the emotions joy and sadness, so called “basic emotions” that show up in facial, vocal and bodily expressive behaviors (Russell, 1980; Ekman, 1992; Jack et al., 2014). Both emotions will be referred to in their broadest sense, i.e., as representing their respective families of emotion (e.g., Scherer et al., 1991; Ekman, 1992). Thus, joy also encompasses emotions like happiness and elation, whereas sadness comprises the emotion of grief. Despite the fact that they are considered in this broad sense, joy and sadness still represent distinct, polar emotions with markedly different phenomenological qualia (Schmitz, 1969; Demmerling and Landweer, 2007; also see Kraxenberger and Menninghaus, 2016a,b). Also, and most importantly for this study, joy and sadness are commonly understood to be universally available (Ekman and Cordaro, 2011) and to serve a communicative function, for instance in terms of the emotional prosody of an utterance.

Emotional prosody is predominantly determined by the suprasegmental features of pitch, tempo, and intensity (Alter, 2002), and is conducive to conveying emotions (Paulmann, 2006). Empirical studies on acoustic cues in the expression of emotions have yielded largely consistent results (Scherer, 1986); this holds particularly for the expression and recognition of joy and sadness. Accordingly, several studies have shown that the emotions of joy and sadness can indeed be distinguished by means of acoustic profiles that are dependent on suprasegmental parameters. In the case of joy, most studies have reported higher values for the mean F0 (pitch) and mean intensity, as well as a faster speech rate. The expression and recognition of sadness, by contrast, has been associated with lower values for these measures (cf. Kaiser, 1962; Van Bezooijen, 1984; Scherer, 1986, 2013; Banse and Scherer, 1996; Ververidis and Kotropoulos, 2006; Pell et al., 2009; Stolarski, 2015).

If joy and sadness can already be reliably detected by means of certain suprasegmental features in simple sentences and utterances, it is reasonable to conjecture that these parameters of emotional prosody should also yield a pronounced effect in poetry—a literary genre widely considered to be eminently emotional (e.g., Hegel, 1986; Winko, 2003; Lüdtke et al., 2014). However, despite the large amount of evidence supporting the idea of acoustic emotional profiles for smaller text units and utterances, the effects of suprasegmental features of emotional prosody on emotion perception in poetry recitation have rarely (if ever) been investigated to date. More importantly, most studies on emotional prosody focused on a bottom-up approach, i.e. they mainly considered acoustic features in relation to an emotional interpretation. The interaction of semantically based emotion content and emotion-supporting prosody, on the other hand, has not been studied before, particularly within poetry. Therefore, we set out and conducted a pre-study on the recitation, i.e., acoustic production of poems, as well as a perception experiment to test for prosody-based sound-emotion associations, each comparing sad and joyful poems.

The first study was designed to validate and establish quantitative measures for emotional prosody in native-speakers' poetry recitation of joyful and sad poems. To this end, participants were asked to read out aloud eight German poems. The suprasegmental features of emotional prosody obtained through this production study served as the basis for manipulating the acoustic presentation of joyful and sad poems in a subsequent perception experiment. This second study was conducted online; it aimed at testing whether and to what extent features of emotional prosody work only in conjunction with semantic understanding or can also influence emotion perception in the absence of semantic access. The study included two experimental conditions in which participants without and with knowledge of German (i.e., English and Japanese without German knowledge, as well as German participants, cf. page 6) were exposed to audio recordings of joyful and sad German poems that were systematically manipulated in terms of emotion-related suprasegmental parameters. In the first condition (Expressive Condition), we presented poems with emotionally expressive prosodies, i.e., a joy-supporting, and a sadness-supporting prosody. In the second condition (Neutral Condition), the same poems were presented with a “neutralized” emotional prosody. Targeting the prosodic features of the poems in this way and comparing participants with and without access to semantic-based emotional content allowed us to examine the relative weights of bottom-up (prosodic) and top-down (semantic) information during emotion perception in poetry.

## Experiment 1

### Materials and methods

#### Participants

Twenty-three participants (15 females; ages ranging from 20 to 32 years, *M* = 25.0, *SD* = 3.1) took part in our study. Inclusion criteria for participation were German as a native language and full legal age. The participants were asked to recite and rate four joyful and four sad poems. All experimental procedures were ethically approved by the Ethics Council of the Max Planck Society and were undertaken with the informed consent of each participant.

#### Poems

We selected eight German poems written in the twentieth century. The poems had between 10 and 16 lines, and featured end rhymes (paired or cross rhymes), as well as a mainly iambic meter. In a previous study (Kraxenberger and Menninghaus, 2016b), four of these poems had been classified as joyful (Werner Bergengruen: *Sommersonett*; Paul Haller: *O leuchtender Septembertag*; Klabund: *Liebeslied, Dein Mund*; Joachim Ringelnatz: *Morgenwonne*), and four as sad (Yvan Goll: *Trauermarsch*; Ferdinand Hardekopf: *Spät*; Else Lasker-Schüler: *Dämmerung*; Jesse Thoor: *Die Zerwartung*). This classification was first based on matching the poems' main themes to phenomenological descriptions of joy and sadness (Schmitz, 1969; Demmerling and Landweer, 2007) and subsequently approved by an analysis of variance. Thus, the poems that were a priori classified as joyful turned out to be rated as significantly more joyful and as less sad than the poems classified as sad (see Kraxenberger and Menninghaus, 2016b).

#### Procedure

For recording their recitations, participants were seated in a sound-attenuated booth. The poems were presented on a screen in a randomized order. Participants were instructed to first read each poem silently. Subsequently, they were asked to rate *how joyful* (hereafter: Joy; German: *freudig*) and *how sad* (hereafter: Sadness; German: *traurig*) they perceived the respective poem to be, using seven-point items ranging from 1 (*not at all*) to 7 (*very much*)^1^. Next, participants were instructed to read the poem aloud. We used a directional headset microphone (DPA, d:fine) and the digital recording device Zoom H4n (sampling rate: 44.1 kHz, amplitude resolution: 16 bits) for recording. All participants read, rated, and recited all eight poems.

For calculating the acoustic measures of pitch and articulation rate, we first annotated each syllable of each recitation for all participants. We used WebMaus (Reichel, 2012; Reichel and Kisler, 2014) for syllabification and for the creation of text-based annotations. In a subsequent step, we controlled syllable boundaries and, if necessary, manually adjusted and corrected them to comply with phonetic conventions regarding phoneme segmentation. We then moved all boundaries to the nearest zero crossing and calculated the syllable rate. In line with Pfitzinger (2001), we analyzed the global net articulation rate, i.e., all actually produced syllables, excluding pauses. Because pauses are known to influence the calculation of speech tempo (cf. Künzel, 1997; Trouvain et al., 2001; Jessen, 2007), and realized pauses during poetry recitation are strongly influenced by verse structure, we decided to exclude all pauses from our analyses of the articulation rate, along with repetitions, corrections, and nonverbal articulations (e.g., coughing). Although literature on acoustic emotion profiles often also report effects of intensity, we decided not to include this parameter in our experiments. This decision was based on the unpredictability of participants' individual tone and volume control, their technical equipment (e.g., sound cards, speaker system, etc.), and their default setting (or adjustment) of volume when participating in our subsequent online study.

#### Analysis

For each participant and poem, we divided the number of actually produced syllables by the total duration of all syllables, excluding pauses. We thereby obtained a value for the number syllables articulated per second for each participant and each recitation. Mean pitch was estimated using the fundamental frequency (F0) analysis in Praat (Boersma and Weenink, 2015). F0 was extracted from the voiced portions of the respective syllables based on an autocorrelation algorithm following the procedure proposed by Hirst (2011). We began with a floor value of 60 Hz and a ceiling value of 700 Hz. We excluded the first and last quartiles from our pitch data to compensate for outliers. Then we calculated F0 again, based on the new floor (minF0) and ceiling (maxF0) values. Potential variance between speakers was taken into account by applying random intercepts to our statistical analyses (see below)^2^.

#### Results

In a first step, we checked whether participants confirmed the preclassification of the poems as joyful or sad. Here we inspected the mean values of the Joy and Sadness ratings. The mean Joy ratings for the poems that were preclassified as joyful were all above the midpoint (4) of the seven-point item measuring Joy (*M* = 5.73, *SD* = 1.19). Likewise, the mean Sadness rating for the poems that were preclassified as sad were all well above the midpoint of the Sadness item (*M* = 5.57, *SD* = 1.20). Additionally, our analyses of variance showed significant differences between participants' ratings for Joy and Sadness of poems that were in line with our preclassification, i.e., poems preclassified as joyful were rated as more joyful and less sad than poems that were preclassified as sad (all *p* ≤ 0.001, all η^2^ ≥ 0.77).

To test whether the mean pitch of the participants' recitations was related to the perceived Joy and Sadness of the poems, we applied linear mixed models with random intercepts for poems and participants and the fixed effects of the participants' ratings for Joy and Sadness. Given a highly significant, negative correlation between the ratings for Joy and Sadness (Pearson Correlation, two-tailed, *r* = −0.91, *p* ≤ 0.001), we included the ratings for Joy and Sadness in separate models. We also included participants' gender as an additional fixed effect (for an overview of gender effects in speech production see, for instance, Titze, 2000).

The results showed that participants' ratings of Joy were significantly related to the mean pitch at which they recited the poems. On average, an increase of joyfulness ratings by one unit on the 7 point scale corresponded to a 1.68 Hz increase in pitch (*SE* = 0.19, *t* = 8.69, *p* ≤ 0.001). Similarly, Sadness ratings significantly predicted the mean pitch of the recitations; here, an increase of sadness ratings by one unit corresponded to a 1.58 Hz decrease in pitch (*SE* = 0.23, *t* = −6.97, *p* ≤ 0.001). The same analysis was applied to test whether the articulation rate (i.e., number of syllables articulated per second) could likewise be predicted by the participants' ratings for Joy and Sadness. The results showed a relation of participants' Joy ratings and the tempo at which they recited the poems by trend (b = 0.02, *SE* = 0.01, *t* = 1.76, *p* ≤ 0.10). The participants' ratings for Sadness showed a significant relation to the articulation rate (*b* = −0.03, *SE* = 0.01, *t* = −2.30, *p* ≤ 0.05), indicating a decrease in tempo when ratings for Sadness increased.

The validation experiment thus provided us with mean pitch and mean rate changes per unit of Joy/Sadness rating. We subsequently used these values for our acoustic manipulations of joy-supporting and sadness-supporting prosody.

## Experiment 2

To test whether emotional prosody can be viewed as a pivotal factor *independent* of the semantic content of acoustically presented poems, and to investigate the role of semantic access with respect to participants' emotion ratings, we designed and conducted a cross-language study. Based on our validation of the suprasegmental features of emotional prosody during poetry recitation as reported in Experiment 1, we included two conditions in our survey (a so-called “Expressive Condition” and a “Neutral Condition”). In the Expressive Condition, we presented each joyful and sad poem with both joy- as well as with sadness-supporting prosodic cues, and hence in a semantic-matching and a semantic-mismatching variant with divergent prosody. In the Neutral Condition, we substantially reduced and hence “neutralized” the prosodic cues. Further, our design allowed us to test the effect of emotional prosody orthogonally to the participants' access to the poems' semantic content (German-speaking vs. non-German-speaking listeners), including, amongst others, syntactic cues, word frequency, or familiarity with German poetry. Our hypotheses and expectations were as follows:

(a) The joy- and sadness-supporting prosodies in the Expressive Condition should substantially affect the emotion ratings of the non-German-speaking listeners who have no linguistic access to the poems' semantic content. More specifically, we expected a main effect of joy- and sadness supporting prosodies that would not interact with the preclassification of the poems as joyful or sad.
(b) In the case of matching prosodic characteristics and emotional content (i.e., joyful poems presented with a joy-supporting prosody), the prosodic distinction between sad and joyful poems should be particularly pronounced. Therefore, participants without semantic access should be able to distinguish between joyful and sad poems without understanding their content.
(c) For the German-speaking listeners in the Expressive Condition, ratings should be strongly influenced by the preclassification of the poems as being joyful or sad in content. In an exploratory manner, we tested whether the potential effects of emotional prosody could be overridden by the semantic access of the German participants. Again, only a main effect of joy- and sadness supporting prosodies without interaction with the preclassification of the poems as joyful or sad would be considered as a pivotal factor.
(d) In the absence of relevant prosodic cues in the Neutral Condition, the participants' ratings should depend only on the poems' semantic content; here, the emotion ratings of the German-speaking and non-German-speaking participants were expected to differ strongly from each other given that non-German speaking participants had no semantic access to the content of the poems.
(e) German-speaking participants in the Neutral Condition were again expected to rate the poems in accordance to our preclassification of the poems as either joyful or sad.

In an exploratory manner, we tested in a first step across all conditions for differences in the ratings of English and Japanese participants (see page 6) that might be due different culture- or language-dependent stances and attitudes toward emotions, as well as due to the different language families English and Japanese are part of. We also tested for differences in the ratings of English and Japanese participants with and without experience in analyzing linguistic audio-material. The latter analyses were based on the assumption that such experience might influence the handling and evaluation of semantically incomprehensible audio material.

### Materials and methods

#### Recordings of audio stimuli

A professional actor recited the eight poems that were used in the validation study. During the recording session, the actor was instructed to read the poems in a rather restrained prosodic manner—i.e., without extreme modulations of voice, timbre, pitch, or tempo—and to avoid an excessive emphasis on metrical regularity and rhyme schemes.

To control for loudness differences between the individual stimuli, we decided to use a normalization based on the recommendation ITU-R BS.1770-3 (International Telecommunication Union, 2012). We used the open source tool R128GAIN (Belkner, 2014) to normalize each stimulus to a loudness value of −23LUFS (absolute measurement) according to R128^3^.

#### Manipulation of suprasegmental features of emotional prosody

In order to assess the relative contribution that emotional prosody and the participants' access to the semantic content of the poems might make to the emotion ratings, we manipulated suprasegmental features of emotional prosody. All manipulations were done using Praat (Boersma and Weenink, 2015); they affected the mean pitch and tempo of the recordings. We also manipulated the poems' mean range of pitch to eliminate outliers with regard to single pitch points.

##### Neutralized prosody

Before modifying the emotional prosody of our recordings in a joy- or sadness-supporting way, we first calculated the mean pitch across all eight recordings of the poems (*M* = 146.70 Hz, *SD* = 7.30, *min* = 135.82 Hz, *max* = 158.24). Then the mean pitch for all recorded poems was set to this mean. The mean pitch range was also adjusted accordingly (*M* = 353.43 Hz, *min* = 75.017 Hz, *max* = 428.445 Hz). Thus, the manipulated recordings had the same mean values for pitch and pitch range but retained their original pitch contours. In this way, we produced recordings that had a natural-sounding human voice with controlled and less variable pitch values than the original recordings.

##### Joy- and sadness-supporting prosodies

**Figure 1:**
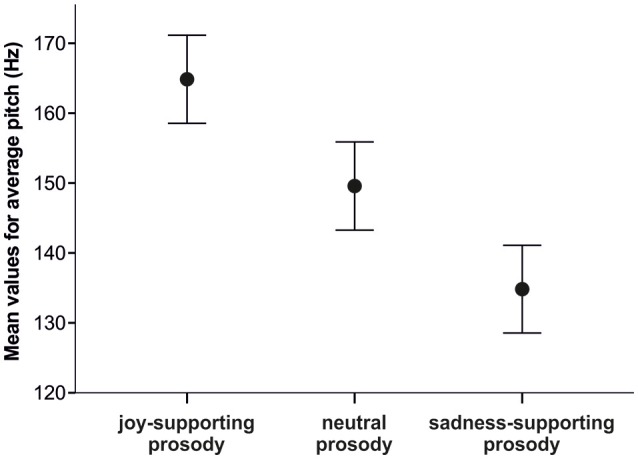
Chart showing mean values of averaged pitch values in Hertz for the three prosodic versions (joy-supporting prosody, neutral prosody, sadness-supporting prosody). Error bars indicate the 95% confidence intervals.

All subsequent pitch manipulations for our first experimental condition (Expressive Condition) were based on the neutral baseline obtained through the changes reported above. Since participants had shown an average pitch increase of 1.68 Hz per unit of joy rating and an average decrease of 1.58 Hz per unit of sadness rating in the validation study, we decided to change pitch values accordingly. To arrive at pitch levels associated with joy and sadness, we increased and decreased each pitch point by 15 Hz (about 10 times the changes per rating level within the validation study), respectively, starting from the neutral pitch levels (for an illustration of the mean pitches in each prosodic version, see Figure 1).

In addition to the pitch changes, we also changed the tempo of each recording of a poem with joy-supporting prosody by decreasing its length factor by 10%. For the emotionally expressive versions of the sad poems (i.e., sadness-supporting prosody), we applied the reverse procedure. That is, we lowered pitch points by 15 Hz and reduced the tempo by 10%. The versions that were to contain mismatching cues of emotional prosody were produced by applying the expressive emotional features of the sad poems to the joyful poems and vice versa. As a result, joyful poems were reduced in pitch and tempo, whereas sad poems were increased in pitch and tempo. Thus, we obtained two versions of each poem, one with an expressive, semantic-matching prosody (i.e., in line with our preclassification of the poems as either joyful or sad), and one with a reverse, mismatching emotional prosody (i.e., contrary to the preclassification).

In order to investigate the influence of semantic content independent of prosodic cues, we used the “neutralized” baseline versions as stimuli for a second condition (Neutral Condition). In this condition, acoustic cues should be rather uninformative regarding emotional prosody, and we expected that German participants' ratings for Joy and Sadness would rely predominantly on their semantic access to the poems. Participants without knowledge of German, however, should have neither semantic nor prosodic cues guiding their emotion ratings.

#### Participants

Native German, English, and Japanese speakers were recruited through postings on diverse mailing lists, through a distribution of postcards, and via social media sites. The inclusion criterion for participation in the study was being of full legal age. The participants did not receive monetary or other compensation. As for the validation study, the Ethics Council of the Max Planck Society ethically approved all experimental procedures. Prior to filling out the online questionnaire, participants provided their written informed consent. Non-German speakers were asked to indicate their German language skills using the level descriptions from the *Common European Framework of Reference for Languages* (CEFR). Participants with German-language skills at level B1 (Threshold) and above were excluded from the survey. All other participants were instructed regarding the procedure of the study.

Two hundred seventy-two participants completed the survey. Of these, 205 were German (137 indicated female gender and 9 participants did not specify their gender; ages ranged from 18 to 46 years, *M* = 32.6 years, *SD* = 8.10). Thirty-five complete data sets were obtained from participants who indicated that English was their mother tongue (21 participants indicated female gender and two participants did not specify their gender; ages ranged from 19 to 45 years, *M* = 33.8 years, *SD* = 7.88; 48.6 % indicated to have some kind of experience with analyzing linguistic audio material). Thirty-two complete data sets were obtained from native Japanese-speaking participants (22 participants were female and 10 were male; ages ranged from 19 to 45 years, *M* = 32.6 years, *SD* = 9.70; 9.4% indicated to have some kind of experience with analyzing linguistic audio material).

Among the native German-speaking group, 100 participants were assigned to the Expressive Condition and 105 participants to the Neutral Condition. Similarly, 16 native English speakers were assigned to the Expressive Condition and 19 to the Neutral Condition. Among the Japanese speakers, 18 were assigned to the Expressive Condition and 14 to the Neutral Condition. The assignment of participants to one of the two conditions was randomized.

#### Online questionnaire and procedure

Ratings were collected using an online survey (in German, English, and Japanese)^4^. Participants were instructed to use headphones and to complete the survey alone without interruption. To become familiar with the rating procedure, participants listened to a practice poem and rated it. Afterwards, they were randomly assigned to one of the two conditions of the survey.

Participants assigned to the Expressive Condition were presented with two joyful and two sad poems with joy-supporting prosody, and two joyful and two sad poems with sadness-supporting prosody. We randomized the order and prosodic version of the poems per participant and made sure that participants would hear a poem only in one version. Participants in the neutral condition were exposed to all eight “neutralized” poems in randomized order. All audio files were stereo recordings with a 44.1 kHz sampling rate and 32 bit amplitude resolution.

After listening to a poem, participants were asked to indicate *how joyful* and *how sad* (German: *freudig/traurig*; Japanese: 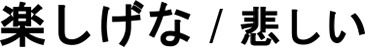)^5^ they perceived the poems to be on a scale ranging from 1 (*not joyful*/*not sad at all*) to 7 (*very joyful*/*very sad*). Additionally, they rated the poems on a bipolar affect item ranging from 1 (*negative*) to 7 (*positive*) (for the distinction between emotion and affect, see Russell, 2003; Scherer, 2005). Ratings were gathered via seven-part visual analog scales (VASs; e.g., Aitken, 1969; Funke and Reips, 2012), allowing a fine-grained measurement of the participants' responses. In addition to language skills and age, we also surveyed whether or not participants' had any experience with the analysis of linguistic audio material. The average duration for survey completion was 17.36 min (*SD* = 6.13, *min* = 10.63, *max* = 49.6).

In each condition, participants' ratings for Joy, Sadness, and Negativity/Positivity correlated highly with one another (Pearson Correlation, two-tailed, all |*r*| ≥ 0.82, all *p* ≤ 0.001). Thus, the bipolar item did not seem to provide substantially different information. For this reason, we exclusively report participants' ratings for Joy and Sadness (hereafter: emotion ratings), in line with the preceding validation study.

## Results

### Ratings of english- and japanese-speaking participants

In order to test whether English- and Japanese speaking participants differed in their emotion ratings, we applied analyses of variance (ANOVAs) to test for potential group differences. Results showed no significant differences between the ratings of English- and Japanese-speaking participants for the Expressive Condition (ratings for Joy: *M*_English_ = 3.81, *SD*_English_ = 1.27, *M*_Japanese_ = 3.87, *SD*_Japanese_ = 1.35, *F*_(1, 270)_ = 0.14, *p* = 0.71, $\eta_{p}^{2}$ = 0.001; ratings for Sadness: *M*_English_ = 4.16, *SD*_English_ = 1.33, *M*_Japanese_ = 4.27, *SD*_Japanese_ = 1.39, *F*_(1, 270)_ = 0.46, *p* = 0.50, $\eta_{p}^{2}$ = 0.002). The same was the case for the Neutral Condition (ratings for Joy: *M*_English_ = 3.73, *SD*_English_ = 1.17, *M*_Japanese_ = 3.81, *SD*_Japanese_ = 1.26, *F*_(1, 262)_ = 0.26, *p* = 0.61, $\eta_{p}^{2}$ = 0.001; ratings for Sadness: *M*_English_ = 4.01, *SD*_English_ = 1.15, *M*_Japanese_ = 4.25, *SD*_Japanese_ = 1.24, *F*_(1, 262)_ = 2.61, *p* = 0.11, $\eta_{p}^{2}$ = 0.01).

In addition, we monitored for differences between participants with and without experience in analyzing linguistic audio-material. Applying again analyses of variance, results showed no significant differences between the ratings of Japanese-speaking participants with and without experience within the Expressive Condition (ratings for Joy: *M*_experienced_ = 3.56, *SD*_experienced_ = 0.84, *M*_unexperienced_ = 3.89, *SD*_unexperienced_ = 1.29, *F*_(1, 18)_ = 0.50, *p* = 0.48, $\eta_{p}^{2}$ = 0.003, ratings for Sadness: *M*_experienced_ = 3.27, *SD*_experienced_ = 0.86, *M*_unexperienced_ = 3.40, *SD*_unexperienced_ = 1.40, *F*_(1, 18)_ = 0.07, *p* = 0.80, $\eta_{p}^{2}$ = 0.000). The same was the case for English-speaking participants with and without analyses-experience in the Expressive Condition (ratings for Joy: *M*_experienced_ = 3.78, *SD*_experienced_ = 1.27, *M*_unexperienced_ = 3.82, *SD*_unexperienced_ = 1.39, *F*_(1, 16)_ = 0.40, *p* = 0.85, $\eta_{p}^{2}$ = 0.000; ratings for Sadness: *M*_experienced_ = 3.91, *SD*_experienced_ = 1.62, *M*_unexperienced_ = 3.73, *SD*_unexperienced_ = 1.32, *F*_(1, 16)_ = 0.42, *p* = 0.52, $\eta_{p}^{2}$ = 0.003). Analyses of the Neutral Condition likewise showed no significant differences between experienced and unexperienced Japanese and/or English participants (all *p* ≥ 0.29, $\eta_{p}^{2}$ ≥ 0.010). Comparing in a further step all Non-German speaking participants (i.e., English and Japanese participants) with and without experience in analyzing linguistic audio-material, results again showed no significant differences, this applied to both the Expressive and the Neutral Condition (all *p* ≥ 0.15, $\eta_{p}^{2}$ ≥ 0.008).

Based on these results, we henceforth considered English- and Japanese-speaking participants in all subsequent analyses jointly as non-German-speaking participants.

#### Statistical analyses

We performed linear mixed effect analyses using the package lme4 (Bates et al., 2014) in the statistical software R (R Core Team, 2013) to predict emotion ratings, i.e., participants' ratings for Joy and Sadness. *P*-values were obtained using lmerTest (Kuznetsova et al., 2015). All other analyses were conducted in SPSS (IBM SPSS Statistics for Windows, Version 22.0, IBM Corp., 2013).

For all models, we included the factors *language* (non-German-speaking participants vs. German-speaking participants) and *poem preclassification* (i.e., the poems' preclassification as either joyful or sad), together with the random intercept factors *participant* and *poem*. In addition, the Expressive Condition allowed us to include the within-participant factor *prosody* (joy-supporting vs. sadness-supporting). In a second step, we replaced this additional factor by *match/mismatch* (match vs. mismatch between prosodic features and semantic content e.g., poems that were preclassified as joyful were presented with a sadness-supporting prosody). The initial models were always specified as full factorial, i.e., as including all possible interaction terms. Subsequent models focused on specific effects when qualified by significant interactions.

#### Expressive condition

##### Full model

The models that were used to predict Joy ratings showed main effects of *poem preclassification* (*b* = 3.53, *SE* = 0.27, *t* = 13.20, *p* ≤ 0.001) and of *language* (*b* = 1.35, *SE* = 0.15, *t* = 8.92, *p* ≤ 0.001). Further, analyses revealed an interaction between *poem preclassification* and *language* (*b* = −2.77, *SE* = 0.21, *t* = −13.27, *p* ≤ 0.001). Importantly, there was no main effect of *prosody* (*b* = 0.12, *SE* = 0.10, *t* = 1.11, *p* = 0.27), but a significant interaction of *prosody* and *language* (*b* = 0.41, *SE* = 0.21, *t* = 1.98, *p* = 0.05). The prediction of participants' Sadness ratings showed the same results. Again, we found main effects for *poem preclassification* (*b* = −3.07, *SE* = 0.28, *t* = −11.03, *p* ≤ 0.001) and *language* (*b* = −1.14, *SE* = 0.18, *t* = −6.23, *p* ≤ 0.001), as well as an interaction of *poem preclassification* and *language* (*b* = 2.10, *SE* = 0.24, *t* = 8.84; *p* ≤ 0.001), and of *prosody* and *language* (*b* = −0.52, *SE* = 0.24, *t* = −2.20, *p* = 0.03). Again, there was no main effect of *prosody* (*b* = −0.15, *SE* = 0.12, *t* = −1.27, *p* = 0.20).

Replacing the factor *prosody* by *match/mismatch*, Joy rating showed main effects of *poem preclassification* (*b* = 3.41, *SE* = 0.27, *t* = 12.76, *p* ≤ 0.001) and of *language* (*b* = 1.76, *SE* = 0.15, *t* = 11.68, *p* ≤ 0.001). We found an interaction between *poem preclassification* and *language* (*b* = −3.19, *SE* = 0.21, *t* = −15.26, *p* ≤ 0.001). *Match/mismatch* showed no main effect (*b* = −0.12, *SE* = 0.11, *t* = −1.11, *p* = 0.27), but a significant interaction with *language* (*b* = −0.41, *SE* = 0.21, *t* = 1.98, *p* = 0.05).

The model predicting Sadness ratings revealed a main effect for *poem preclassification* (*b* = −2.92, *SE* = 0.28, *t* = −10.48, *p* ≤ 0.001) and *language* (*b* = −1.66, *SE* = 0.18, *t* = −9.09, *p* ≤ 0.001), as well as an interaction of *poem preclassification* and *language* (*b* = 2.62, *SE* = 0.24, *t* = 11.05, *p* ≤ 0.001). The factor *match/mismatch* showed no main effect (*b* = 0.52, *SE* = 0.12, *t* = 1.27, *p* = 0.20), but a significant interaction with the factor *language* (*b* = 0.52, *SE* = 0.24, *t* = −2.20, *p* = 0.03; for decompositions of these interactions, see below).

##### Non-german-speaking participants

The decomposition of the interactions per language-group (Non-German-speaking vs. German-speaking participants) revealed that both emotion ratings of the non-German-speaking participants were significantly influenced by the factor *prosody* (ratings for Joy: *b* = 0.46, *SE* = 0.22, *t* = 2.11, *p* = 0.04; ratings for Sadness: *b* = −0.63, *SE* = 0.22, *t* = −2.83, *p* = 0.01). Also, analyses showed an effect of *poem preclassification* (ratings for Joy: *b* = 0.68, *SE* = 0.22, *t* = 3.11, *p* ≤ 0.01; ratings for Sadness: *b* = −0.91, *SE* = 0.26, *t* = −3.44, *p* = 0.01).

When replacing *prosody* by *match/mismatch*, analyses showed that the emotion rating of the non-German participants showed a significant effect of *match/mismatch* (ratings for Joy: *b* = −0.46, *SE* = 0.22, *t* = −2.11, *p* = 0.04; ratings for Sadness: *b* = 0.63, *SE* = 0.22, *t* = 2.83, *p* = 0.01), but not of *poems preclassification* (ratings for Joy: *b* = 0.22, *SE* = 0.22, *t* = 1.00, *p* = 0.32; ratings for Sadness: *b* = −0.27, *SE* = 0.26, *t* = −1.04, *p* = 0.06).

##### German-speaking control group

In contrast to the results for the non-German speaking participants, native German-speaking listeners' ratings were only significantly influenced by the factor *poem preclassification* (ratings for Joy: *b* = 3.52, *SE* = 0.34, *t* = 10.71, *p* ≤ 0.001; ratings for Sadness: *b* = −3.06, *SE* = 0.32, *t* =, *p* ≤ 0.001). They rated joyful poems as more joyful (*N* = 400, *M* = 5.49, *SD* = 1.09) than sad poems (*N* = 400, *M* = 2.05, *SD* = 0.98, *F*_(1, 798)_ = 2196.22, *p* ≤ 0.001, η^2^ = 0.73) and sad poems as sadder than joyful ones (*M* = 5.27, *SD* = 1.34, *F*_(1, 798)_ = 1,821.48, *p* ≤ 0.001, η^2^ = 0.59). The presentation of joyful and sad poems with either a joy- or a sadness-supporting prosody did not significantly affect the emotion ratings of the German-speaking listeners (ratings for Joy: *b* = −0.17, *SE* = 0.13, *t* = −1.28, *p* = 0.20; ratings for Sadness: *b* = 0.09, *SE* = 0.16, *t* = 0.57, *p* = 0.57).

Analyses of the models that included the factor *match/mismatch* instead of *prosody* revealed that German-speaking participants' emotion ratings were not influenced by the factor *match/mismatch* (ratings for Joy: *b* = −0.11, *SE* = 0.09, *t* = −1.18, *p* = 0.20; ratings for Sadness: *b* = 0.15, *SE* = 0.11, *t* = 1.31, *p* = 0.20). Rather, their ratings showed, again, an effect of *poem preclassification* (ratings for Joy: *b* = 3.41, *SE* = 0.33, *t* = 10.37, *p* = ≤ 0.001; ratings for Sadness: *b* = −2.91, *SE* = 0.32, *t* = −9.05, *p* = ≤ 0.001).

#### Neutral condition

##### Full model

As in the Expressive Condition, the results for the Neutral Condition showed a main effect of *poem preclassification* (ratings for Joy: *b* = 3.36, *SE* = 0.28, *t* = 11.90, *p* ≤ 0.001; ratings for Sadness: *b* = −3.03, *SE* = 0.24, *t* = −12.41, *p* ≤ 0.001) as well as *language* (ratings for Joy: *b* = 1.28, *SE* = 0.11, *t* = 11.26, *p* ≤ 0.001; ratings for Sadness: *b* = −0.83, *SE* = 0.14, *t* = −5.83, *p* ≤ 0.001) on participants' emotion ratings. Further, our analyses again revealed interactions between these two independent variables (ratings for Joy: *b* = −2.64, *SE* = 0.14, *t* = −18.38, *p* ≤ 0.001; ratings for Sadness: *b* = 2.28, *SE* = 0.16, *t* = 13.94, *p* ≤ 0.001).

**Figure 2:**
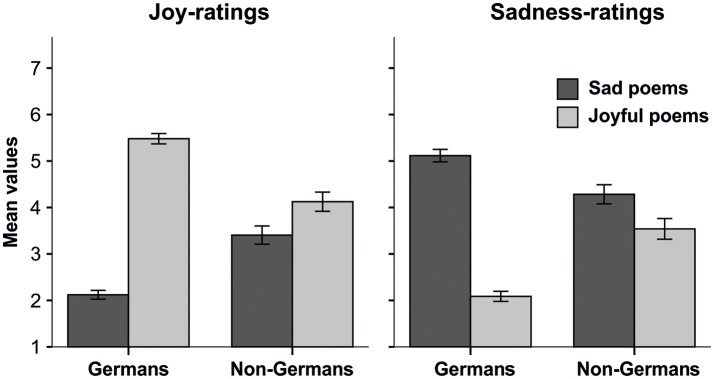
Bar charts showing mean values of Joy and Sadness- ratings from German and non-German speaking participants, separately displayed for joyful and sad poems. Error bars indicate the 95% confidence intervals.

The decomposition of the interaction between *poem preclassification* and *language* showed that the non-German-speaking participants clearly differed in their ratings from the German-speaking group. A separate inspection of the mean values of emotion rating for the joyful and sad poems showed that non-German-speaking participants rated joyful poems as less joyful (*M* = 4.15, *SD* = 1.20) and sadder (*M* = 3.54, *SD* = 1.29) than the German-speaking group (*M*_Joy_ = 5.48, *SD*_Joy_ = 1.16, *M*_Sadness_ = 2.09, *SD*_Sadness_ = 1.13, all *F*_(1, 549)_ ≥ 134.42, all *p* ≤ 0.001, all η^2^ ≥ 0.20). Likewise, the non-German-speaking participants rated sad poems as more joyful (*M* = 3.41, *SD* = 1.14) and less sad (*M* = 4.28, *SD* = 1.20) than the German-speaking participants (*M*_Joy_ = 2.12, *SD*_Joy_ = 0.99, *M*_Sadness_ = 5.12, *SD*_Sadness_ = 1.40, all *F*_(1, 550)_ ≥ 37.95, all *p* ≤ 0.001, all η^2^ ≥ 0.07; see Figure 2 for an illustration using the mean values for emotions ratings of German and non-German speaking participants for poems preclassified as joyful and sad).

##### Non-german-speaking participants

The factor of *poem preclassification* again showed an influence on the ratings of the non-German-speaking listeners (ratings for Joy: *b* = 0.72, *SE* = 0.17, *t* = 4.10, *p* = 0.01; ratings for Sadness: *b* = −0.74, *SE* = 0.21, *t* = −3.47, *p* = 0.05).

##### German-speaking control group

The effect of *poem preclassification* held for the German-speaking listeners in the Neutral Condition. Therefore, joyful poems were rated as more joyful, and sad poems were rated as sadder (ratings for Joy: *b* = 3.36, *SE* = 0.35, *t* = 9.66, *p* ≤ 0.001; ratings for Sadness: *b* = −3.03, *SE* = 0.29, *t* = −10.58, *p* ≤ 0.001).

## Discussion

In line with the hypothesis of prosody-driven sound-emotion associations in poetry, we found a significant relation between prosodic features of participants' recitations of poems and their ratings of joy and sadness: On average, pitch and tempo values were higher for joyful than for sad poems across German native speakers (Experiment 1). Our cross-language perception experiment (Experiment 2) further illustrated that pitch and tempo values are indeed interpreted with respect to emotional content: non-German-speaking participants without semantic access to the poems were particularly sensitive to the prosodic manipulation, i.e., they rated poems with a joy-supporting prosody as more joyful and poems with a sadness-supporting prosody as sadder. Further, our analyses confirmed the expectation that, in the Expressive Condition, non-German-speaking participants were sensitive to the difference between poem versions in which emotional content and prosodic features matched, and poems in which emotional content and prosodic features did not match. At the same time, however, German participants did not show significantly different ratings dependent on our prosodic manipulations. Rather, the content-based preclassification of the poems as either joyful or sad was the only significant predictor of their ratings, independently of whether prosodic features were in line with the semantic content of the poems or not. Thus, in addition to the results of our experiments, our investigation also highlights the importance of semantic access to the content of a poem, which, if available, has to be considered the most vital predictor of emotion perception in poetry.

In the neutral condition, as expected, non-German-speaking participants' and German-speaking-participants' ratings differed significantly. However, and rather unexpectedly, the content-based preclassification of the poems as joyful or sad also predicted the emotion ratings of the non-German-speaking listeners in the neutral condition, albeit to a weaker degree than for the German control group. Given the result from the Expressive Condition that non-German-speaking listeners were sensitive to the difference between match and mismatch of prosodic features and semantic content, the correlation between preclassification and emotion ratings of the non-German group is likely not to be based on linguistic comprehension. Rather, the likelihood of non-German-speaking participants to detect the correct emotion may rather derive from the fact that the presented poems retained prosody-based characteristic of joy and sadness beyond the parameters investigated here. As stated in the Stimulus section, the presented poems retained their original pitch contours and we exclusively defined and modified emotional prosody in terms of mean pitch and mean articulation rate. Clearly, these measures hide a large amount of important variance, and they cannot account at all for the dynamic contours of emotional prosody throughout intonation phrases, which are likely to make a strong contribution to the overall perception of emotional prosody (e.g., Pierrehumbert, 1980; Gussenhoven, 2004; Ladd, 2008).

The non-inclusion of the pitch contour characteristics in the experimental modification constitutes a general limitation of our study. Future studies are thus called for that not only consider pitch contour and additional cues of emotional prosody, but also investigate suprasegmental features separately and in a step-wise addition. Also, further evidence is needed to examine prosodic effects and their influence on emotion perception during poetry reading by native recipients and to investigate to what extend content-based emotion perception also relies on prosodic feature during (silent) reading. Furthermore, future studies should include stimuli that cannot as clearly be assigned to dominant emotions as it was the case for the poems used in our experiments. Additional variables could include poetic forms such as sound poems, or mixed emotions such as nostalgia or melancholy.

Notwithstanding these limitations, our study offers a prosody-based account for sound-emotion associations in poetry that goes beyond the repeatedly and inconsistently tested assumption of a relation between phoneme frequencies and emotion perception. Our results strongly indicate that emotional prosody plays a substantial role in sound-emotion associations in poetry, if content-based semantic access is unavailable. The suprasegmental features investigated here are an important factor for recitations by native speakers, and also influence the emotion ratings of listeners who have no access to the content of the presented poems. Thus, emotional prosody is an important dimension in both expressing and deciphering the emotional content of poems. This is all the more important since purely semantic understanding may be not as straightforward in poetry as in other textual forms, be that for reasons of the greater importance of stylistic figures, deviations from common language use, the particularly condensed forms of evoking the content, or more associative imagery.

## Author contributions

MK designed the study and compiled the poetic corpus. MK and AR decided upon experimental conditions, manipulated the audio files and gathered data. MK conducted all analyses. MK, WM, and MS interpreted the data and wrote the paper.

### Conflict of interest statement

The authors declare that the research was conducted in the absence of any commercial or financial relationships that could be construed as a potential conflict of interest.

## Footnotes

^1^Some additional items from the questionnaire were not considered in the analyses presented here.

^2^For a description of the packages and statistical software used, see pages 6–7.

^3^R128 uses a loudness range to measure the variation of loudness on a macroscopic time scale in loudness units (LUs, relative measurement), where one LU corresponds to one Db. (For a more detailed description and the computation of the loudness range as a measurement of loudness levels, see Broadcasting Service (Sound) Series, 2012).

^4^The survey was administered online between March 10 and July 25, 2016, using the software (Unipark QuestBack Globalpark AG., 2014). Some additional items from the questionnaire were not considered in the analyses presented here.

^5^We used the following phrases in the German and Japanese versions of our questionnaire: *Wie freudig ist das Gedicht? / Wie traurig ist das Gedicht?*  Please note that none of the participants commented negatively on the comprehensibility of our questionnaire.
